# Is Noncycloplegic Photorefraction Applicable for Screening Refractive Amblyopia Risk Factors?

**Published:** 2012-01

**Authors:** Zhale Rajavi, Hiva Parsafar, Alireza Ramezani, Mehdi Yaseri

**Affiliations:** Ophthalmic Research Center, Shahid Beheshti University of Medical Sciences, Tehran, Iran

**Keywords:** Photorefraction, Cyclorefraction, Amblyopia, Screening

## Abstract

**Purpose:**

To compare the accuracy of noncycloplegic photorefraction (NCP) with that of cycloplegic refraction (CR) for detecting refractive amblyopia risk factors (RARFs) and to determine cutoff points.

**Methods:**

In this diagnostic test study, right eyes of 185 children (aged 1 to 14 years) first underwent NCP using the PlusoptiX SO4 photoscreener followed by CR. Based on CR results, hyperopia (≥ +3.5 D), myopia (≥ −3 D), astigmatism (≥ 1.5 D), and anisometropia (≥ 1.5 D) were set as diagnostic criteria based on AAPOS guidelines. The difference in the detection of RARFs by the two methods was the main outcome measure.

**Results:**

RARFs were present in 57 (30.8%) and 52 (28.1%) of cases by CR and NCP, respectively, with an 89.7% agreement. In contrast to myopia and astigmatism, mean spherical power in hyperopic eyes was significantly different based on the two methods (P < 0.001), being higher with CR (+5.96 ± 2.13 D) as compared to NCP (+2.37 ± 1.36 D). Considering CR as the gold standard, specificities for NCP exceeded 93% and sensitivities were also acceptable (≥ 83%) for myopia and astigmatism. Nevertheless, sensitivity of NCP for detecting hyperopia was only 45.4%. Using a cutoff point of +1.87 D, instead of +3.5 D, for hyperopia, sensitivity of NCP was increased to 81.8% with specificity of 84%.

**Conclusion:**

NCP is a relatively accurate method for detecting RARFs in myopia and astigmatism. Using an alternative cutoff point in this study, NCP may be considered an acceptable device for detecting hyperopia as well.

## INTRODUCTION

Refractive errors are one of the most important causes of amblyopia.^1^,^2^ Amblyopia affects 2 to 5% of the population^1^,^3^ and is the leading cause of unilateral decreased vision among children and young adults in central Europe.^1^

Due to the importance of early diagnosis and treatment, different methods of amblyopia screening have being assessed for preverbal and preschool-age children.^4^ Some of these methods detect amblyopia directly by measuring visual acuity (VA) while others do so indirectly by checking refractive errors and ocular deviation.^5^ The Vision in Preschoolers study showed that retinoscopy without cycloplegia had the highest sensitivity for amblyopia screening, followed by the Welch-Allyn autorefractor, the Retinomax autorefractor (Nikon), and vision screening using Lea Symbols in decreasing order with constant specificity of 90%.^6^,^7^

The gold standard for detecting refractive errors in children is cycloplegic refraction.^1^ However, its application in screening programs is limited because it requires an experienced examiner and is time consuming.^8^,^9^ In addition, the use of cycloplegic drops may predispose the child to untoward side effects. Cycloplegic autorefraction is a faster method; nonetheless, it requires considerable cooperation and is therefore not practical in some children.^1^,^8^

Photorefraction is a technique that can measure refractive errors without administering cycloplegia even in very young subjects and hence simplifies amblyopia screening. In a very short time, it simultaneously assesses both eyes for refractive errors, pupil size, inter-pupillary distance, deviations, ptosis, and media opacities, while maintaining the same amplitude of accommodation in both eyes.^2^ It has an appealing appearance which attracts attention and fixation by using red targets and playing music. However, certain disadvantages may limit its use. It has been shown that pupil size (< 3 and ≥ 8 mm), media haziness, fixation problems, and some posterior segment pathologies may affect the results. In addition, this measurement is limited to refractive errors ranging from −7 to +5 diopters.

Since there is a wide range of reported sensitivities and specificities (63% to 94% and 62% to 99%, respectively)^1^ for photorefractors in the literature, we conducted this study to compare noncycloplegic photorefraction (NCP) with cycloplegic refraction, which is considered as the gold standard. The results of this study may also provide practical cutoff points for defining refractive amblyopia risk factors (RARFs) when photorefractors are employed for screening.

## METHODS

This diagnostic study included all children 1 to 14 years of age who were examined at the eye clinic at Imam Hossein Medical Center, Tehran, Iran. Cases with mental retardation, impaired fixation, strabismus, ptosis, and any other organic ophthalmic disorder interfering with refraction were excluded.

The study was approved by the review board/ethics committee of the Ophthalmic Research Center, Shahid Beheshti University of Medical Sciences, Tehran, Iran. Before recruitment, the study protocol was explained to all parents and informed consent was obtained in accordance with ethical standards of the declaration of Helsinki.

All subjects underwent a preliminary ophthalmic examination using a pen light, direct ophthalmoscope, and slit lamp. In the first step, NCP was performed in both eyes using the PlusoptiX SO4 photoscreener (PlusoptiX GmbH, Nürnberg, Germany) by an independent and experienced technician in a quiet location and under room lighting. To run the PlusoptiX SO4, we used an electrical power source (220v, ∼AC), a monitor, a keyboard, and a mouse. A printer was connected to the system to provide a printout for each case. The only parameter used for the purpose of this study was refractive error of the right eyes. In subjects diagnosed with anisometropia by either method, data for left eyes was also considered. In case of out-of-range measurements, as shown on the photoscreener printouts, the uppermost limits of the photorefractor (−7 or +5) were considered for analysis.

In the cycloplegic refraction step, cycloplegia was achieved by instillation of cyclopentolate 1% and tropicamide 1% eye drops within a 5-minute interval. After 45 minutes, cycloplegic autorefraction was performed using a Topcon autorefractometer (RM-8800; Topcon Medical, Oakland, NJ, USA) by a masked and experienced optometrist. If the child was uncooperative for autorefraction, cycloplegic retinoscopy would be performed instead. We excluded children in whom one or both methods of cycloplegic refraction were not feasible. Ophthalmic examinations were completed for all cases afterwards and detected abnormalities were managed accordingly.

Based on the results of cycloplegic refraction, hyperopia ≥+3.5 D, myopia ≥ −3 D, with or against the rule astigmatism ≥ −1.5 D, oblique astigmatism ≥ 1 D, and anisometropia ≥ 1.5 D (sphere or cylinder) were set as criteria for defining significant refractive errors. These values were based on the American Academy of Pediatric Ophthalmology and Strabismus (AAPOS) uniform guidelines^10^ to compare the two methods employed in this study. Astigmatism was recorded and evaluated in minus cylinder notations. If the axis of astigmatism was within 10 degrees on either side of the horizontal or vertical axes, it was defined as with or against-the-rule astigmatism, respectively; otherwise, it was considered as oblique astigmatism.

### Statistical Methods

Results are presented in mean, standard deviation, frequency, and percentages. To describe the agreement of measurements, we employed Pearson correlation. We used paired t-test to evaluate differences between the two methods. In order to find the best cutoff points, we utilized a ROC curve. Agreement between the methods was evaluated using preset criteria and also with newly defined cutoff points by utilizing sensitivity, specificity, positive predictive values, negative predictive values, number of false positives, number of false negatives, and overall agreement, as well as the Kappa index. In all evaluations, cycloplegic refraction was considered as the gold standard. All statistical analyses were performed using the SPSS software version 17 (SPSS Inc., Chicago, IL, USA).

## RESULTS

A total of 191 children were recruited. NCP was not possible in 3 children (1.6%) due to small pupil size in one, and poor cooperation in two other subjects. Cycloplegic refraction could not be performed in 3 other (1.6%) uncooperative children. After excluding these cases, results for 185 right eyes of 185 children including 92 (49.7%) male and 93 (50.3%) female subjects with mean age of 5.87 ± 2.33 (range, 1 to 14) years were analyzed and reported. Of these, 80% of subjects were younger than 7 years. Cycloplegic refraction was performed using an autorefractometer in 167 eyes (90.3%), and by retinoscopy in 18 eyes (9.7%). The upper or lower limit of the photorefractor was considered for analysis in 6 eyes (3.2%), which had an out-of-range response. Of these, 4 eyes were hyperopic and 2 were myopic.

Overall, at least one RARF was present in 52 (28.1%) and 57 (30.8%) eyes as determined by NCP and cycloplegic refraction, respectively, with an overall agreement of 89.7%. RARFs were detected using NCP and cycloplegic refraction in 15 (8.1%) and 22 (11.9%) eyes in the hyperopic range (≥ +3.5 D), 5 (2.7%) and 6 (3.2%) eyes in the myopic range (≥ −3 D), 46 (24.9%) and 43 (23.2%) eyes in the astigmatic range (≥ 1.5 D), as well as 21 (11.8%) and 22 (12.4%) eyes with anisometropia (≥ 1.5 D), respectively. The highest disparity was noted in detecting hyperopia (Table 1). With-the-rule and oblique astigmatism were detected by NCP in 33 (17.8%) and 31 (16.8%) eyes and by cycloplegic refraction in 28 (15.1%) and 26 (14.1%) eyes, respectively. None of the eyes had significant (≥ 1.5 D) against-the-rule astigmatism by either method.

**Figure 1.**
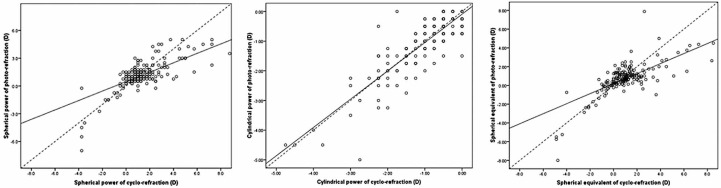
Pearson correlation between noncycloplegic photorefraction and cyclorefraction for spherical error, cylindrical error and spherical equivalent (r=0.76, r=0.86, r=0.76, respectively).

The mean ± standard deviations for spherical power, cylindrical power, and spherical equivalent measured by NCP (+1.15 ± 1.77, −0.99 ± 0.96, and +0.71 ± 1.76 D, respectively) were significantly correlated with those measured by cycloplegic refraction (+1.45 ± 2.48, −0.94 ± 0.86, and +0.98 ± 2.52 D, respectively). The corresponding Pearson correlations were 0.75, 0.86, and 0.76, respectively (P < 0.001 for all comparisons; Fig. 1).

Mean values of spherical and cylindrical power by NCP and cycloplegic refraction were compared (Table 1) in each category of hyperopia (≥ +3.5 D), myopia (≥ −3 D) and astigmatism (≥ −1.5 D). Mean spherical power of eyes in the hyperopic range was significantly different as determined by the two methods (P < 0.001), being higher with cycloplegic refraction than with NCP. However, differences in mean spherical power in myopic eyes and mean cylindrical power in eyes with astigmatism were not statistically significant (Table 1).

Considering cycloplegic refraction as the gold standard, sensitivity, specificity and other accuracy parameters of NCP were calculated in the various ranges of refractive errors used for amblyopia screening (Table 2). NCP had acceptable specificity (93 to 100%) for detecting all types of refractive errors, and acceptable sensitivity (83 to 85%) for myopia and astigmatism; however, its sensitivity for detecting hyperopia (≥ +3.5 D) was only 45.4%. Accordingly, overall agreement between the two methods was better for myopia (99.5%) than astigmatism (91.3%) and hyperopia (90.8%).

**Figure 2.**
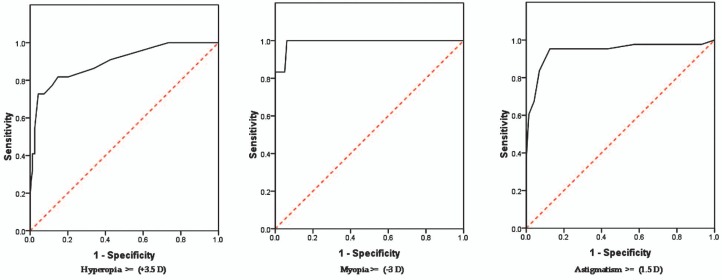
ROC curves for noncycloplegic photorefraction in detecting hyperopia ≥ +3.5 D, myopia ≥ −3.0 D, and astigmatism ≥ 1.5 D. Areas under the curve were 89.6%, 99.1%, and 94.2%, respectively.

In order to find the best cutoff points for NCP in amblyopia screening for all types of refractive errors, ROC curve analysis was applied; this revealed that for myopic errors, powers calculated by NCP were the same as those obtained by cycloplegic refraction. For hyperopia however, the appropriate cutoff for NCP was +1.87 D to detect errors ≥ +3.5 D as detected by cycloplegic refraction. For astigmatism, the suitable cutoff point was −1.12 D for detection of errors ≥ −1.5 D as determined by cycloplegic refraction (Fig. 2).

## DISCUSSION

Although cycloplegic refraction remains the gold standard for detecting refractive errors^1^, NCP has been introduced as a method for screening RARFs in infants and children.^1^,^8^ In children under the age of 3 or 4 years, a normal NCP result does not always imply normal visual acuity.^11^ For amblyopia screening however, it has been shown that the sensitivity, specificity and positive predictive values of photorefraction are higher than those of visual acuity tests in non-verbal children. In addition, compared to visual acuity tests, NCP in children can be performed relatively faster and does not need any prior learning.^3^,^12^

In our study, RARFs were detected in 52 (28.1%) and 57 (30.8%) of studied children as determined by NCP and cyclorefraction, respectively. Corresponding figures were reported to be 53% and 67% by Matta et al.^13^ The overall agreement between the two methods was found to be 89.7% in our study which is comparable to those obtained in other studies, i.e. 84%^14^ and 94%^10^.

In the present study, significant Pearson correlation coefficients of 0.76, 0.86 and 0.76 were observed between NCP and cyclorefraction for spherical, cylindrical and spherical equivalent refractive errors respectively. Comparable correlation coefficients (0.63, 0.70, and 0.63, respectively) were reported between the same two methods by Erdurmus et al^8^.

In our study, a similar number of significant myopic and astigmatic refractive errors were detected by the two methods; nonetheless, NCP identified fewer hyperopic eyes. This is not an unexpected finding due to preservation of accommodative capacity during NCP leading to 2.68 D of myopic shift in hyperopic eyes. This shift was reported to be 0.7 D in the study by Erdurmus et al^8^. Comparing NCP in children with photorefraction wearing +3.00 D glasses, Schaeffel et al^11^ demonstrated myopic shift of 2.40 D with the latter method. They reported that children with higher hyperopia had greater amounts of myopic shift. The authors however concluded that the use of +3.00 D glasses is not an accurate method for detecting hyperopia by photorefraction.

Schimitzek and Lagreze^9^ noted 0.96 D of difference in spherical equivalent refractive error using photorefraction with and without cycloplegia in patients in a wide age range of 2 to 81 years. In their study, some patients did not accommodate while others accommodated up to 4 D. They also noticed that performing cycloplegic photorefraction might lead to incorrect measurement of astigmatic power and axis due to peripheral aberrations caused by pupillary dilation. They recommended photorefraction without cycloplegia to accurately measure cylindrical power and axis, followed by cycloplegic photorefraction to detect the amount of spherical power. If we had used cycloplegic instead of noncycloplegic photorefraction in our study, we might have found greater correlation between the two methods in hyperopic eyes. However, we believe that using cycloplegic eye drops is not convenient in amblyopia screening programs targeting large numbers of children.

The discordant amounts of myopic shift in some of the above mentioned studies and ours may be due to different methodology, versions/models of photoscreeners, patient age, and range of hyperopia.

For myopic and astigmatic errors, measurements obtained by NCP in our study are reliable and may be used with acceptable sensitivity without modification in screening programs. This is comparable to findings reported by Schaeffel et al^11^. In the hyperopic range, nonetheless, lower values of spherical power measured by NCP should be set as a cutoff point for screenings purposes. Applying a modified cutoff value, i.e. +1.87 D instead of +3.5 D as suggested by ROC curve analysis in our study, increases the sensitivity of NCP from 45.4% to 81.8% for detecting hyperopia hence making it suitable for detecting all types of refractive error in children.

The sensitivity and specificity of noncycloplegic photorefraction in comparison to cyclorefraction have been reported to be variable, ranging from 63% to 94% and 62% to 99%, respectively.^1^ In addition, the sensitivity and predictive value of NCP have been reported to be higher than visual acuity testing.^14^ This is comparable to observed values of sensitivity for NCP in our study only for myopia (83.3%) and astigmatism (85.7%), but not for hyperopia. In the latter group, sensitivity was low (45.4%) emphasizing the need for a group analysis in such studies. Measured specificities for NCP in our study were 96.9%, 100%, and 85.7% for hyperopia, myopia, and astigmatism, respectively. We should stress however, that conclusions regarding coefficient values in myopia should be made with caution because of the limited number of cases in this subgroup.

The feasibility of a diagnostic test is very important in children. NCP was not possible in 3 cases (1.6%) in our study. Similarly, cycloplegic refraction could not be performed for three (1.6%) other subjects. Cycloplegic autorefraction was not possible in 18 children (9.7%) who underwent manual cyclorefraction which is operator-dependant and time consuming. These findings emphasize the better applicability of noncycloplegic photorefraction in children. In the study by Erdurmus et al^8^, photorefraction could not be performed in 1.5% of subjects; this was due to pupil size < 3 mm or > 8 mm, limited range of the photorefractor, and peripheral lens aberrations through dilated pupils.

In the present study, two independent technicians performed either of the diagnostic methods, adding the advantage of masking. We were obliged to perform manual cyclorefraction in children who were uncooperative for cycloplegic autorefraction. This changed an automated measurement to a technician-based measurement which could be a potential source of error. Additionally, we included children from a wide age range (1 to 14 years) to improve generalizability of our results; however when accommodation is presumed to be a major confounding factor, difference in age might affect the results. Small sample size in myopic group and lesser amount of astigmatism (up to 1.5 D) in our cases, were also other limitations of our study.

In summary, based on our study, NCP can be an appropriate method for amblyopia screening in children and detecting risk factors for refractive amblyopia. It can be performed relatively rapidly and is applicable for the vast majority of children. The only point one should consider is that the optimal cutoff point for hyperopia may be different from that used in cycloplegic refraction. We recommend applying the following cutoff points to increase precision. Our suggested values for NCP-based amblyopia screening are +2.0, −3.0, and 1.25 D, for detecting hyperopia ≥ +3.5 D, myopia ≥ −3.0 D, and astigmatism ≥ 1.5 D based on cycloplegic refraction. These findings need to be confirmed in future studies on a larger sample focusing on age-stratified groups.

## Figures and Tables

**Table 1.** Comparison of refractive amblyopia risk factors between the two methods

		**Spherical power**		**Cylindrical power**	
		**Hyperopia (≥ +3.5 D)**	**Myopia (≥ −3 D)**	**WTR/ATR (≥ −1.5 D)**	**Oblique (≥ − 1 D)**
Cyclorefraction	n (%)	22 (11.9)	6 (3.2)	28 (15.1)	26 (14.1)
	mean ± SD	+5.96 ± 2.13	−5.63 ± 4.72	−2.21 ± 0.80	−1.76 ± 0.84
Noncycloplegic Photorefraction	n (%)	15 (8.1)	5 (2.7)	33 (17.8)	31 (16.8)
	mean ± SD	+2.37 ± 1.36	−4.38 ± 2.27	−2.44 ± 0.97	−1.56 ± 1.08
Difference	95% CI	2.68 (1.74 to 3.62)	−1.25 (−6.44 to −3.94)	0.22 (−0.03 to −0.47)	−0.20 (−0.47 to −0.06)
P-value		< 0.001	0.563	0.077	0.127

D, diopter; WTR, with the rule; ATR, against the rule; n, number; SD, standard deviation; CI, confidence interval

**Table 2.** Agreement coefficient values for noncycloplegic photorefraction compared to standard cyclorefraction together with the calculated cutoff points based on ROC curve analysis

**Power in Diopters**	**Spherical power**		**Cylindrical power (≥ −1.50)**	**Any refractive amblyopia risk factor**
	**Hyperopia ≥ +3.50**	**Myopia ≥ −3.00**	**≥ −1.50**	**Sphere ≥ +3.50 or ≥ −3.00 or Cylinder ≥ −1.50**
Frequency	22	6	43	57
Sensitivity (%)	45.4	83.3	85.7	79.0
Specificity (%)	96.9	100	93.0	94.5
Positive predictive value (%)	66.7	100	78.3	86.5
Negative predictive value (%)	92.9	99.4	95.7	91.0
True positive	10	5	36	45
True negative	158	179	132	121
False positive	5	0	10	7
False negative	12	1	7	12
Overall agreement	90.8	99.5	91.3	89.7
Prescreening prevalence	11.9	3.2	23.2	30.8
Kappa index	0.492	0.906	0.762	0.753
Cutoff points (Diopters)	1.87	−3.0	±1.12	-
Area under the curve	89.6	99.1	94.2	-
Sensitivity for cutoff points (%)	81.8	83.3	95.3	91.2
Specificity for cutoff points (%)	84.7	100	87.4	82.0
Positive predictive value (%)	41.9	100	69.5	69.3
Negative predictive value (%)	97.2	99.4	99.2	95.4
True positive	18	5	41	52
True negative	138	179	125	105
False positive	25	0	18	23
False negative	4	1	1	5
